# Supplementary material for: Mechanisms of extracellular electron transfer in anaerobic methanotrophic archaea
Source: Nat Commun. 2024 Feb 17;15:1477. doi: 10.1038/s41467-024-45758-2 (PMC10874420; doi:10.1038/s41467-024-45758-2)
Supplement: Supplementary file 1 — Supplementary Information [file 41467_2024_45758_MOESM1_ESM.pdf]

Supplementary Information

Mechanisms of extracellular electron transfer in anaerobic methanotrophic archaea

Heleen T Ouboter[1], Rob Mesman[1], Tom Sleutels[2,3], Jelle Postma[4], Martijn Wissink[1], Mike S M Jetten[1], Annemiek ter Heijne[5], Tom Berben[1], Cornelia U Welte[1]*

[1]Department of Microbiology, Radboud Institute for Biological and Environmental Sciences, Radboud University, Heyendaalseweg 135, 6525AJ Nijmegen, the Netherlands

[2]Wetsus, European Centre of Excellence for Sustainable Water Technology, Oostergoweg 9, 8911, MA, Leeuwarden, the Netherlands

[3]Faculty of Science and Engineering, University of Groningen, Nijenborgh 4, 9747, AG, Groningen, the Netherlands

[4]Department of General Instrumentation, Radboud University, Heyendaalseweg 135, 6525AJ Nijmegen, the Netherlands

[5]Environmental Technology, Wageningen University & Research, Bornse Weilanden 9, 6708 WG Wageningen, The Netherlands

*Address correspondence to Cornelia U. Welte, E-mail address: c.welte@science.ru.nl

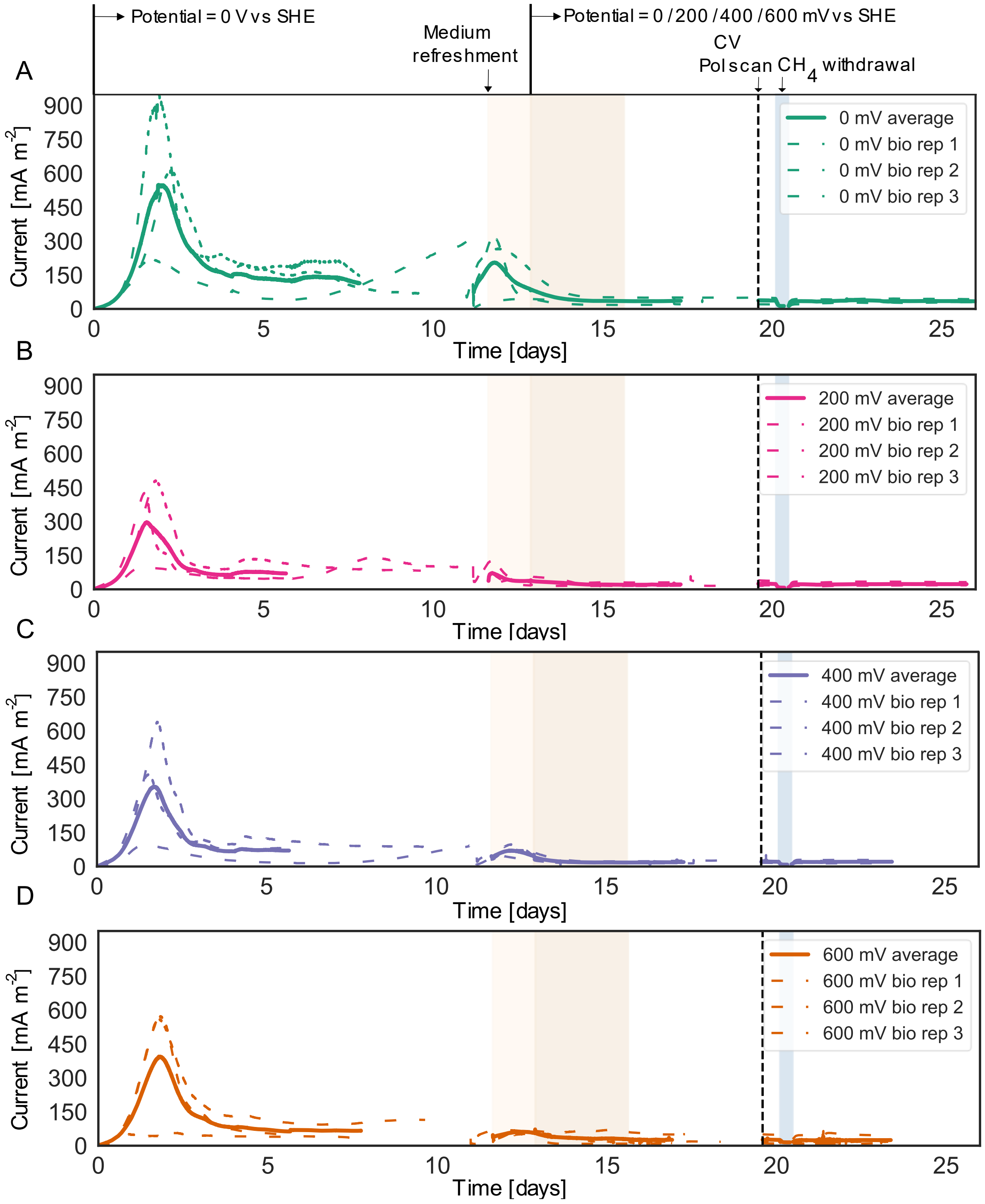

Figure S1: Bioelectrochemical data collected during experiment 1. (A) 0 V vs. SHE condition (green lines); (B) 200 mV vs. SHE condition (pink lines); (C) 400 mV vs. SHE condition (purple lines); (D) 600 mV vs. SHE condition (orange lines). In A-D, two distinct phases are visible: (i) a start-up phase during which all systems were operated at 0 mV versus the standard hydrogen electrode (SHE), represented by the first blank part and the light yellow part of the Figure, with the beginning of the light yellow part indicating the time when the medium was refreshed, and (ii) a subsequent phase during which

the potential was maintained at 0 mV or switched to 200 mV, 400 mV, or 600 mV vs SHE (dark yellow inset and onwards). In this part we conducted cyclic voltammetry scans and polarisation scans, as indicated by the dashed line, and tested for methane-dependent current, depicted by the blue inset. At the end of the experiment, samples were collected for metagenome and metatranscriptome sequencing and the biofilm was visualized using microscopy. Three biological replicates were run for each poised anode condition (dashed lines), with the average of those three systems indicated as solid line. Source data are provided as a Source Data file.

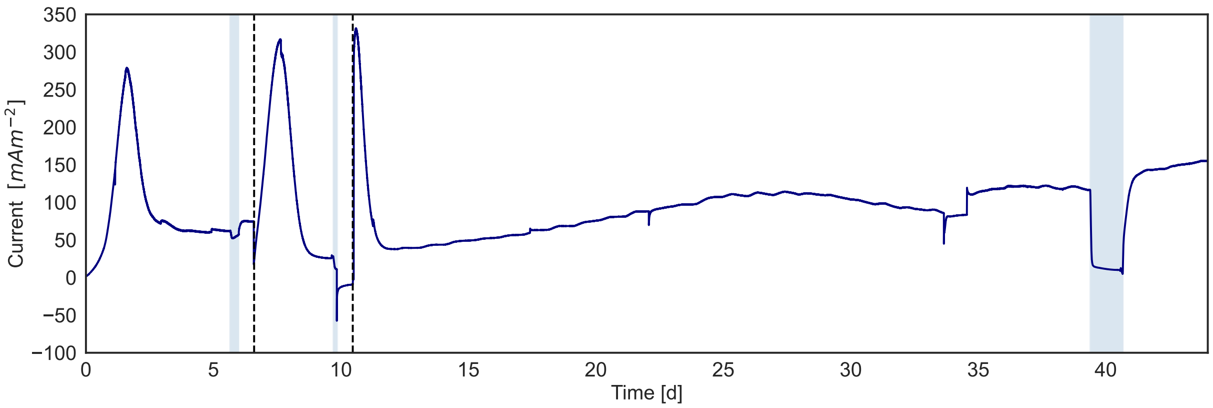

Figure S2: Bioelectrochemical data collected during experiment 2. The microbial community was incubated for 6.5 weeks at 0 mV vs SHE with two medium refreshments as indicated by the dashed lines. Methane-dependent current was measured as indicated by the blue insets. At the end of the experiment, samples were collected for metagenome sequencing. This condition was run with one biological replicate. Source data are provided as a Source Data file.

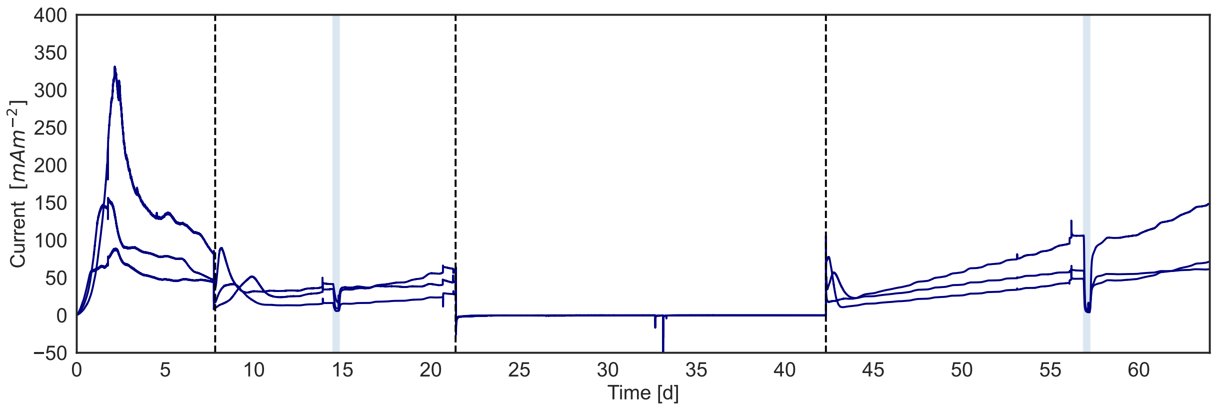

Figure S3: Bioelectrochemical data collected during experiment 3. The microbial community was incubated for 9 weeks at 0 mV vs SHE with three medium refreshments and a three-week famine phase at -400 mV vs SHE occurring between the second and third medium refreshment. The dashed lines represent the medium refreshments, and the famine phase is indicated by the dashed lines. Methane-dependent current was measured before and after the famine phase as indicated by the blue insets of the Figure. At the end of the experiment, the biofilm was visualized using microscopy. This experiment was run with three biological replicates. Source data are provided as a Source Data file.

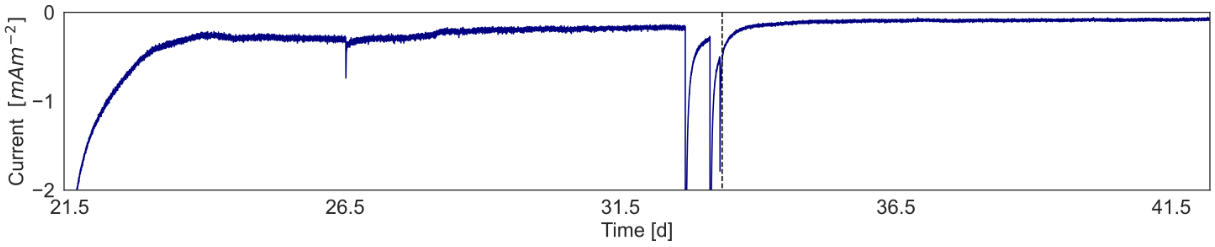

Figure S4: Bioelectrochemical data during the famine phase depicted in Figure S3 in which the potential was -400 mV vs Standard Hydrogen Electrode (SHE). At 33.4 days, the system was closed to measure the production of methane in batch as depicted by the dashed line. This experiment was run with three biological replicates. Source data are provided as a Source Data file.

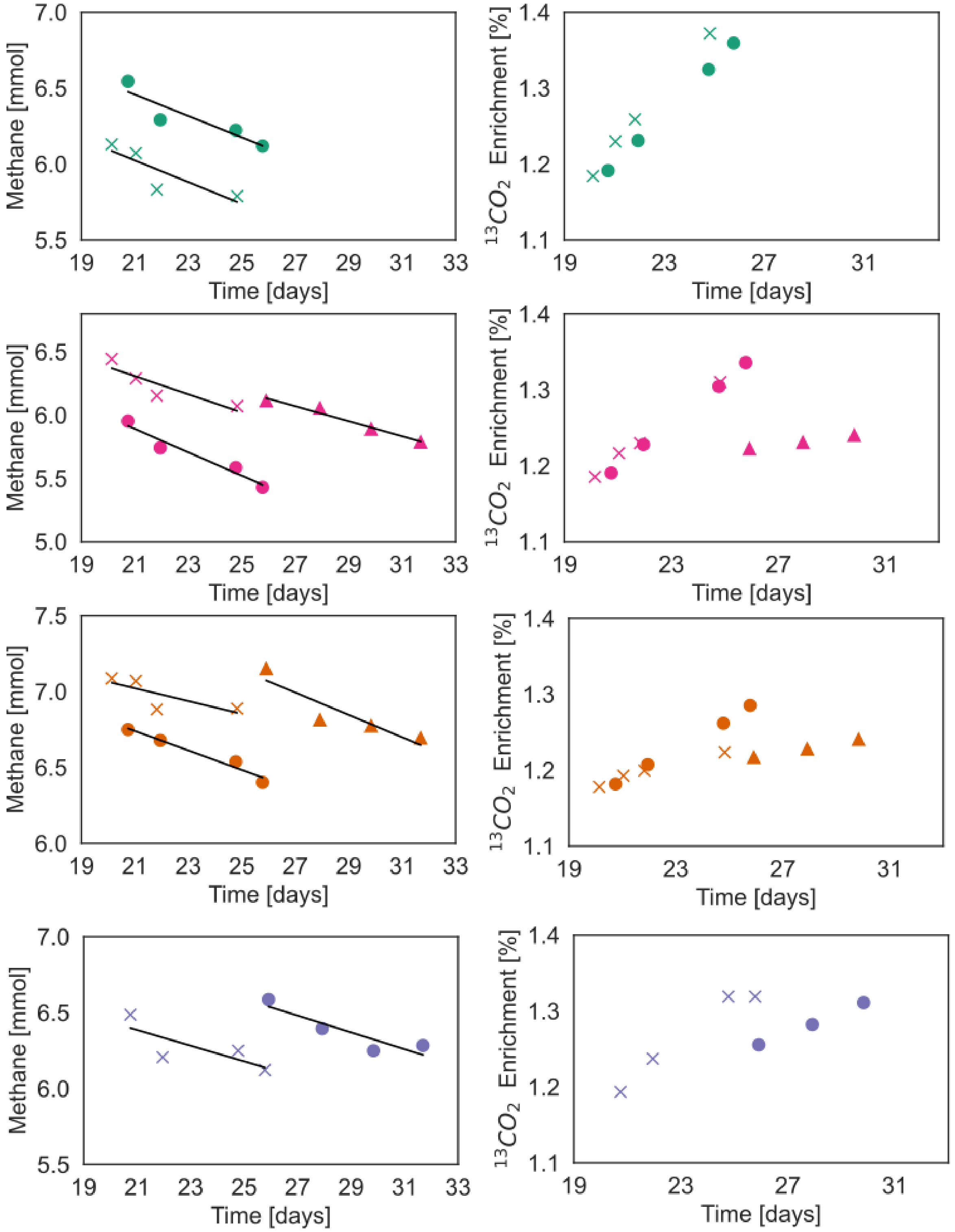

Figure S5: Total methane consumption (left) and relative enrichment of $^{13}CO_2$ (expressed as a percentage) compared to total CO_2 (right) for experiment 1. Green represents 0 mV, pink represents 200 mV, orange represents 400 mV and purple represents 600 mV. The different symbols represent biological replicate bioelectrochemical systems (n=2 for 0 and 600 mV; n=3 for 200 and 400 mV).

These results were collected at the end of the experiment after introducing $^{13}CH_4$ to a sealed system. From this experiment the Coulombic efficiency was calculated to be 5.5% ± 0.013, which was calculated using formula $CE = \frac{Q}{e*n*F}$ with CE coulombic efficiency, Q the cumulative charge expressed in Coulombs (C), the moles of electrons per mole methane, n the moles of methane being consumed and F the Faraday constant being 96485 C mol^{-1}. However, it is challenging to draw conclusions from this measurement due to the large number of samples that was taken over a short period of time increasing the chances for methane to escape and making the calculation inaccurate. Source data are provided as a Source Data file.

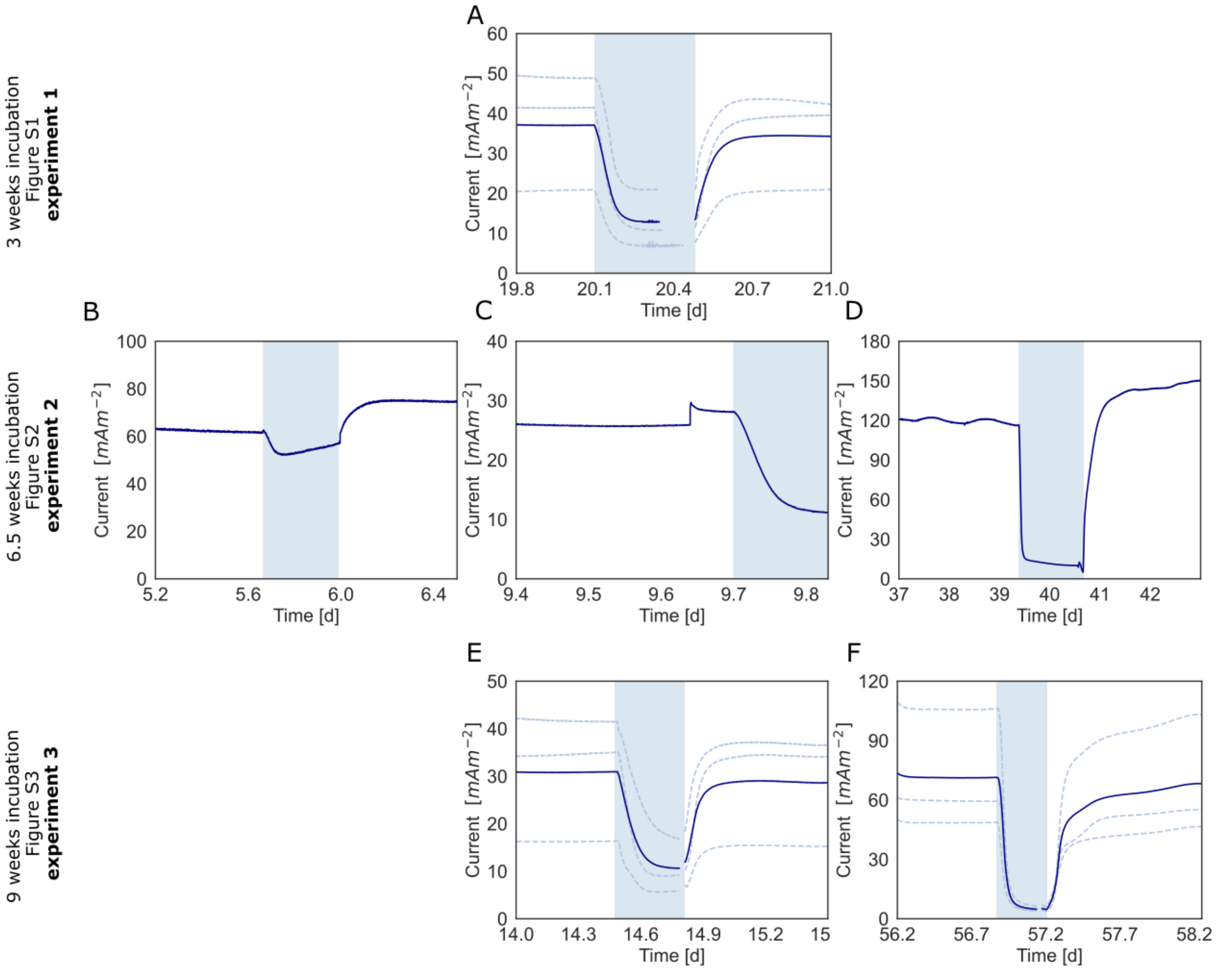

Figure S6: Influence of the number of medium refreshments accompanied by a longer incubation time on the methane-dependent current'. The blue inset indicates where argon was used in the gas phase instead of methane to determine methane-dependent current. This Figure shows the primary

data that is related to Figure 3. Biological replicates are indicated as dashed lines, with solid lines indicating the averages. A, n=3; B, n=1; C, n=1; D, n=1; E, n=3; F, n=3. Source data are provided as a Source Data file.

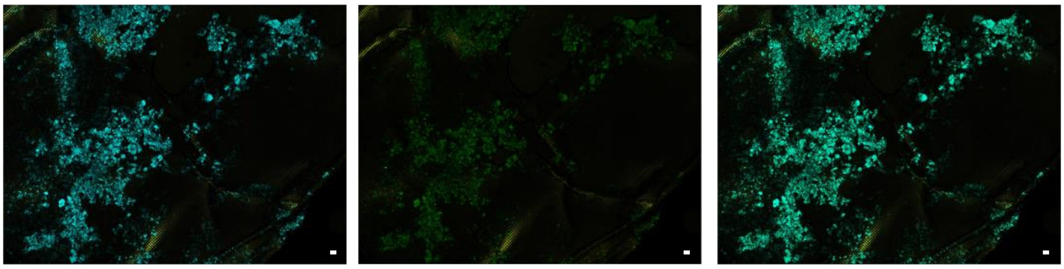

Figure S7: Visualization of the biofilm on the gold electrode using confocal laser scanning microscopy and fluorescence *in situ* hybridization. Archaea are labelled using a FLUOS probe (cyan, left image), '*Ca*. Methanoperedens' using a Cy3 probe (green, middle image), and both micrographs are overlaid in the right image, indicating that no other archaea than '*Ca*. Methanoperedens' are present. The scale bar is 100 µm. The micrographs are chosen as representatives of two independent experiments.

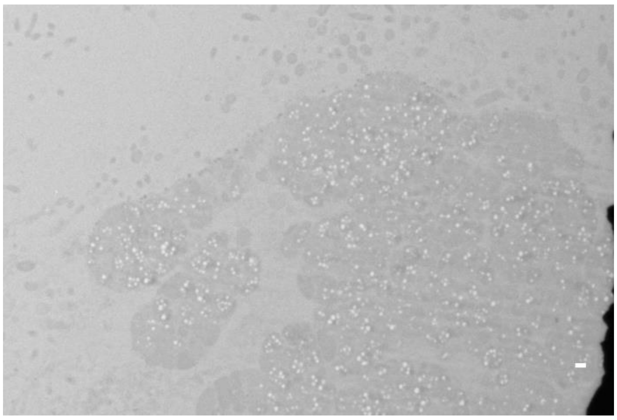

Figure S8: Transmission electron micrograph for the distance estimation of '*Ca*. Methanoperedens' to the electrode. The electrode is visualized in black, with '*Ca*. Methanoperedens' clusters visible in the vicinity. The scale bar is 1 µm. This micrograph is a representative chosen from two independent experiments.

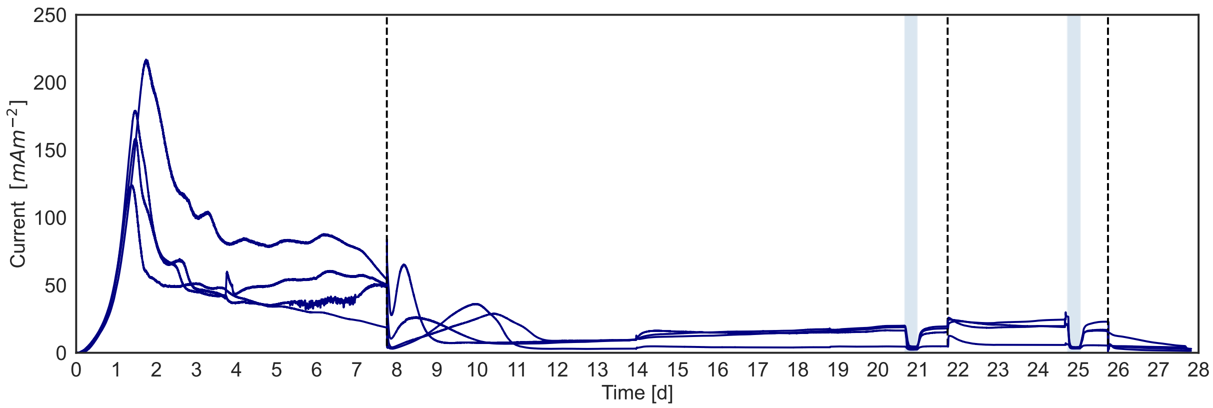

Figure S9: Bioelectrochemical data collected during the experiment in which we tested the influence of a bacterial antibiotic mixture (vancomycin, streptomycin, ampicillin and kanamycin at concentrations of 50 µg mL^{-1} each). Four biological replicates were run. Before and after the addition of antibiotics the methane-dependent current was tested as depicted by the blue insets in which methane was replaced by argon. Near the end of the experiment, depicted by the third dashed line, we tested the influence of 2-bromoethanesulfonate (BES) (20 mM) in two biological replicates (Figure S8) and the influence of the archaeal antibiotic puromycin (50 µg mL^{-1}) affecting the production of ribosomes in two other biological replicates (Figure S9). Medium refreshment is depicted by the first dashed line. Source data are provided as a Source Data file.

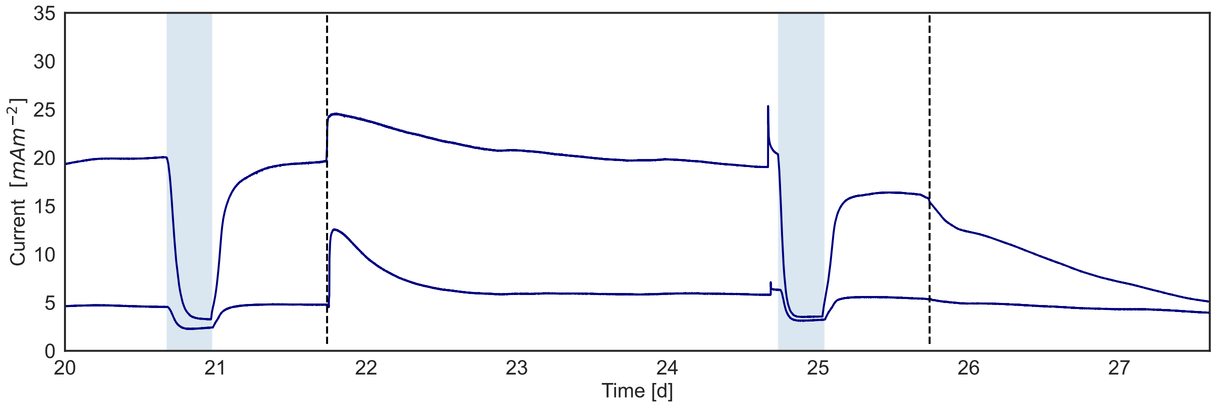

Figure S10: Bioelectrochemical data collected during the experiment in which the influence of antibiotics was tested; enlarged from Figure S9. The first dashed line indicates the addition of the bacterial antibiotics mixture and the second dashed line indicating the addition of archaeal antibiotic

puromycin (50 µg mL^{-1}). The blue inset indicates where argon was used in the gas phase instead of methane. Two biological replicates were run. Source data are provided as a Source Data file.

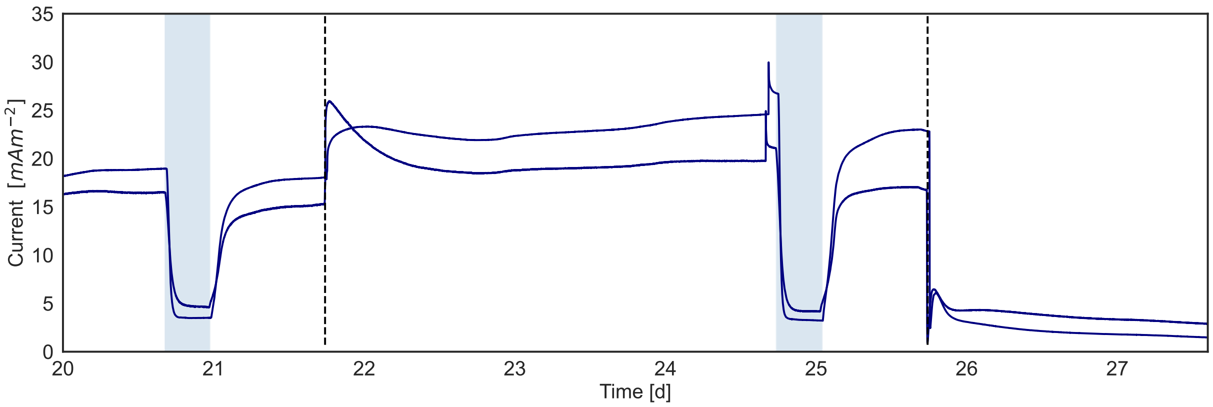

Figure S11: Bioelectrochemical data collected during the experiment in which the influence of antibiotics was tested, enlarged from Figure S7. The first dashed line indicates the addition of the bacterial antibiotics mixture and the second dashed line indicating the addition of MCR inhibitor 2-bromoethanesulfonate (20 mM). The blue part indicates the part where argon was used in the gas phase instead of methane. Two biological replicates were run. Source data are provided as a Source Data file.

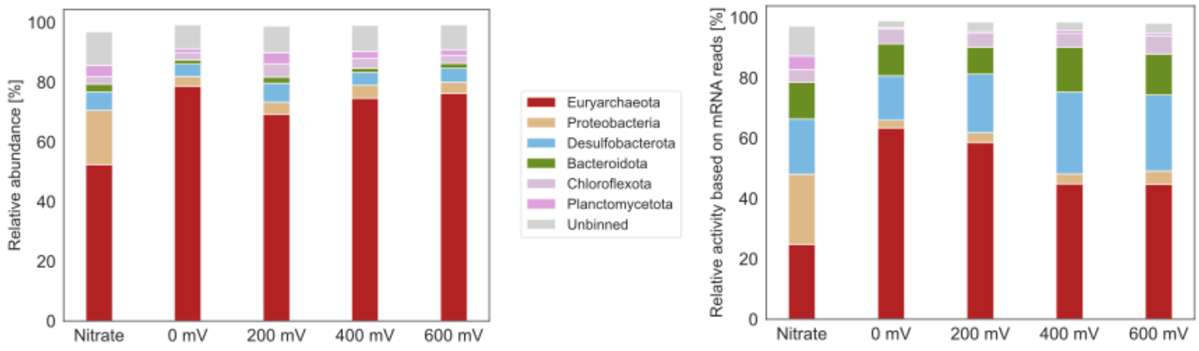

Figure S12: Relative abundance and relative activity at phylum level determined by mapping nucleic acid and mRNA reads to the metagenome assembled genomes (MAGs). The Methanoperedenaceae and Geobacteraceae family both contain only one MAG that was identified as '*Ca*. Methanoperedens' and *Geobacter* sp., respectively, based on the classification by the Genome Taxonomy Database (GTDB) in this study. The completeness of the '*Ca*. Methanoperedens',

Geobacter sp. and Ignavibacteriaceae bin is 99.3%, 99.4% and 97.2% and the contamination of these bins is 5.2%, 0% and 0.6. For these Figures we applied a threshold of 1% relative abundance. RNA samples were collected at 0 mV in triplicate (2 biological, 1 technical replicate), at 200 mV in triplicate (3 biological replicates), at 400 mV in quadruplicate (3 biological replicates, 1 technical replicate), at 600 mV in quadruplicate (3 biological replicates, 1 technical replicate), and under nitrate growth in triplicate (3 biological replicates). The differences in RNA samples for transcriptomics were a result of low amounts of biomass obtained and challenges associated with RNA extraction in archaea. DNA extraction for metagenomics was performed from one bioanode per experimental condition in experiment 1, and once during experiment 2. Source data are provided as a Source Data file.
