# Supplementary material for: Mechanisms of extracellular electron transfer in anaerobic methanotrophic archaea
Source: Nat Commun. 2024 Feb 17;15:1477. doi: 10.1038/s41467-024-45758-2 (PMC10874420; doi:10.1038/s41467-024-45758-2)
Supplement: Supplementary file 3 — Description of Additional Supplementary Files [file 41467_2024_45758_MOESM3_ESM.pdf]

Description of Additional Supplementary Files:

Supplementary Data 1: Overview of all metatranscriptomics data
