# Supplementary material for: Mechanisms of extracellular electron transfer in anaerobic methanotrophic archaea
Source: Nat Commun. 2024 Feb 17;15:1477. doi: 10.1038/s41467-024-45758-2 (PMC10874420; doi:10.1038/s41467-024-45758-2)
Supplement: Supplementary file 5 — Reporting Summary [file 41467_2024_45758_MOESM5_ESM.pdf]

nature portfolio

Corresponding author(s): Cornelia Welte

Last updated by author(s): Jan 17, 2024

Reporting Summary

Nature Portfolio wishes to improve the reproducibility of the work that we publish. This form provides structure for consistency and transparency in reporting. For further information on Nature Portfolio policies, see our Editorial Policies and the Editorial Policy Checklist.

Statistics

For all statistical analyses, confirm that the following items are present in the figure legend, table legend, main text, or Methods section.

n/a	Confirmed	
[ ]	[x]	The exact sample size (*n*) for each experimental group/condition, given as a discrete number and unit of measurement
[ ]	[x]	A statement on whether measurements were taken from distinct samples or whether the same sample was measured repeatedly
[x]	[ ]	The statistical test(s) used AND whether they are one- or two-sided<br>*Only common tests should be described solely by name; describe more complex techniques in the Methods section.*
[x]	[ ]	A description of all covariates tested
[x]	[ ]	A description of any assumptions or corrections, such as tests of normality and adjustment for multiple comparisons
[ ]	[x]	A full description of the statistical parameters including central tendency (e.g. means) or other basic estimates (e.g. regression coefficient) AND variation (e.g. standard deviation) or associated estimates of uncertainty (e.g. confidence intervals)
[x]	[ ]	For null hypothesis testing, the test statistic (e.g. *F*, *t*, *r*) with confidence intervals, effect sizes, degrees of freedom and *P* value noted<br>*Give P values as exact values whenever suitable.*
[x]	[ ]	For Bayesian analysis, information on the choice of priors and Markov chain Monte Carlo settings
[x]	[ ]	For hierarchical and complex designs, identification of the appropriate level for tests and full reporting of outcomes
[x]	[ ]	Estimates of effect sizes (e.g. Cohen's *d*, Pearson's *r*), indicating how they were calculated

Our web collection on statistics for biologists contains articles on many of the points above.

Software and code

Policy information about availability of computer code

Data collection	No software was used for data collection
Data analysis	Only publicly available software and code was used for this study.<br>Metatranscriptomics:<br>sickle 1.33<br>kallisto 0.46.1<br><br>Metagenomics:<br>FastQC 0.11.9<br>BBDUK 37.76<br>MEGAHIT v1.1.1-2-g02102e1<br>BBmap 37.76<br>SAMtools 1.19<br>MaxBin 2.0<br>MetaBAT 2.14<br>CONCOCT 1.1.0<br>BinSanity 0.4.4<br>DAS tool 1.1.2<br>Prodigal

Kraken2 2.1.2
Kaiju 1.7.2
prokka 1.13.3

For manuscripts utilizing custom algorithms or software that are central to the research but not yet described in published literature, software must be made available to editors and reviewers. We strongly encourage code deposition in a community repository (e.g. GitHub). See the Nature Portfolio guidelines for submitting code & software for further information.

Data

Policy information about availability of data

All manuscripts must include a data availability statement. This statement should provide the following information, where applicable:

- Accession codes, unique identifiers, or web links for publicly available datasets
- A description of any restrictions on data availability
- For clinical datasets or third party data, please ensure that the statement adheres to our policy

All DNA and RNA sequencing data have been deposited in the European Nucleotide Archive under BioProject PRJNA995526 (https://www.ebi.ac.uk/ena/browser/view/PRJNA995526). The processed DNA and RNA sequencing data are provided in the Supplemental Data file in this article. The Genome Taxonomy Database used for classification of metagenome-assembled genomes is available under https://gtdb.ecogenomic.org/. The generated bioelectrochemistry and physiology raw data are provided in the Supplementary Information and the Source Data file. Source data are provided with this paper.

Research involving human participants, their data, or biological material

Policy information about studies with human participants or human data. See also policy information about sex, gender (identity/presentation), and sexual orientation and race, ethnicity and racism.

Reporting on sex and gender	no human participants were used in this study
Reporting on race, ethnicity, or other socially relevant groupings	no human participants were used in this study
Population characteristics	no human participants were used in this study
Recruitment	no human participants were used in this study
Ethics oversight	no human participants were used in this study

Note that full information on the approval of the study protocol must also be provided in the manuscript.

Field-specific reporting

Please select the one below that is the best fit for your research. If you are not sure, read the appropriate sections before making your selection.

☒ Life sciences ☐ Behavioural & social sciences ☐ Ecological, evolutionary & environmental sciences

For a reference copy of the document with all sections, see nature.com/documents/nr-reporting-summary-flat.pdf

Life sciences study design

All studies must disclose on these points even when the disclosure is negative.

Sample size	For physiological measurements, biological triplicates were chosen; for transcriptomics, biological triplicates were aimed for.
Data exclusions	No data were excluded
Replication	All bioelectrochemistry experiments were performed in biological triplicate. From these systems, RNA samples were collected at 0 mV in triplicate (2 biological, 1 technical replicate), at 200 mV in triplicate (3 biological replicates), at 400 mV in quadruplicate (3 biological replicates, 1 technical replicate), at 600 mV in quadruplicate (3 biological replicates, 1 technical replicate), and under nitrate growth in triplicate (3 biological replicates). The differences in RNA samples for transcriptomics and bioelectrochemistry experiments were a result of low amounts of biomass obtained and challenges associated with RNA extraction in archaea leading to failure to obtain some samples. DNA extraction for metagenomics was performed from one bioanode per experimental condition in experiment 1, and once during experiment 2.
Randomization	Randomization was not relevant to our study; source material was a microbial bioreactor enrichment and therefore identical for the chosen study conditions.
Blinding	Blinding was not relevant to our study; we did not include any human participants and/or animals. Blinding was also not possible because the analyses were carried out by the person in charge of the experiments.

Reporting for specific materials, systems and methods

We require information from authors about some types of materials, experimental systems and methods used in many studies. Here, indicate whether each material, system or method listed is relevant to your study. If you are not sure if a list item applies to your research, read the appropriate section before selecting a response.

Materials & experimental systems

n/a	Involved in the study
☒	☐ Antibodies
☒	☐ Eukaryotic cell lines
☒	☐ Palaeontology and archaeology
☒	☐ Animals and other organisms
☒	☐ Clinical data
☒	☐ Dual use research of concern
☒	☐ Plants

Methods

n/a	Involved in the study
☒	☐ ChIP-seq
☒	☐ Flow cytometry
☒	☐ MRI-based neuroimaging

Plants

Seed stocks	no seed stocks were used in this study
Novel plant genotypes	no plants were used in this study
Authentication	no plants or seed stocks were used in this study
